# White Matter Changes of Neurite Density and Fiber Orientation Dispersion during Human Brain Maturation

**DOI:** 10.1371/journal.pone.0123656

**Published:** 2015-06-26

**Authors:** Yi Shin Chang, Julia P. Owen, Nicholas J. Pojman, Tony Thieu, Polina Bukshpun, Mari L. J. Wakahiro, Jeffrey I. Berman, Timothy P. L. Roberts, Srikantan S. Nagarajan, Elliott H. Sherr, Pratik Mukherjee

**Affiliations:** 1 Department of Radiology and Biomedical Imaging, University of California San Francisco, San Francisco, California, United States of America; 2 Program in Bioengineering, University of California San Francisco, San Francisco, California, United States of America; 3 Department of Neurology, University of California San Francisco, San Francisco, California, United States of America; 4 Department of Radiology, Children’s Hospital of Philadelphia, University of Pennsylvania, Philadelphia, Pennsylvania, United States of America; Beijing Normal University, CHINA

## Abstract

Diffusion tensor imaging (DTI) studies of human brain development have consistently shown widespread, but nonlinear increases in white matter anisotropy through childhood, adolescence, and into adulthood. However, despite its sensitivity to changes in tissue microstructure, DTI lacks the specificity to disentangle distinct microstructural features of white and gray matter. Neurite orientation dispersion and density imaging (NODDI) is a recently proposed multi-compartment biophysical model of brain microstructure that can estimate non-collinear properties of white matter, such as neurite orientation dispersion index (ODI) and neurite density index (NDI). In this study, we apply NODDI to 66 healthy controls aged 7–63 years to investigate changes of ODI and NDI with brain maturation, with comparison to standard DTI metrics. Using both region-of-interest and voxel-wise analyses, we find that NDI exhibits striking increases over the studied age range following a logarithmic growth pattern, while ODI rises following an exponential growth pattern. This novel finding is consistent with well-established age-related changes of FA over the lifespan that show growth during childhood and adolescence, plateau during early adulthood, and accelerating decay after the fourth decade of life. Our results suggest that the rise of FA during the first two decades of life is dominated by increasing NDI, while the fall in FA after the fourth decade is driven by the exponential rise of ODI that overcomes the slower increases of NDI. Using partial least squares regression, we further demonstrate that NODDI better predicts chronological age than DTI. Finally, we show excellent test—retest reliability of NODDI metrics, with coefficients of variation below 5% in all measured regions of interest. Our results support the conclusion that NODDI reveals biologically specific characteristics of brain development that are more closely linked to the microstructural features of white matter than are the empirical metrics provided by DTI.

## Introduction

The human brain undergoes an extended period of postnatal development that results in sensory, motor, cognitive and behavioral maturation. Improvement of higher cognitive functions, such as executive attention, cognitive control, and working memory, from childhood to adulthood reflects changes in structural and functional brain networks, rather than in isolated brain regions [1]. Histologic studies have shown changes in synaptic density through adolescence [2] [3], and progression of white matter myelination continuing into adulthood [4] [5]. Studies using conventional MR imaging have demonstrated significant brain maturation through childhood, with increases in white matter volume extending past adolescence [6] [7] [8]. Progress in understanding human brain development has been accelerated by the advent of diffusion MR imaging techniques, which are sensitive to microstructural tissue changes. In particular, diffusion tensor imaging (DTI) studies show widespread, nonlinearly increasing white matter anisotropy through childhood, adolescence, and into adulthood [9] [10].

Although DTI has proven useful as a tool for studying brain development during the past two decades, the diffusion tensor remains limited as a basic statistical description of water diffusion within a voxel from images typically acquired at only a single diffusion-weighting factor (*b* value), which represents a single spherical shell in *q*-space. The assumption of Gaussian diffusion that underpins the DTI model also breaks down at *b* values much in excess of 1000 s/mm^2^, whereas the investigation of restricted and strongly hindered diffusion such as within the intracellular space requires higher diffusion-weighting factors. For these reasons, there is not necessarily any direct link between DTI metrics and the underlying tissue architecture. Commonly used DTI measures, such as fractional anisotropy (FA), mean diffusivity (MD), axial diffusivity (AD) and radial diffusivity (RD), may lack the specificity to unravel the distinct microstructural features of gray matter and white matter [11] [12].

With continuing improvements in MR scanner field strength, gradient performance, RF head coil arrays and pulse sequences, it has now become possible to routinely collect multi-shell high angular resolution diffusion imaging (HARDI) including at *b* values much greater than the 1000 s/mm^2^ that has been standard for DTI. Concurrently, there have been advances in biophysical compartmental modeling to directly infer microstructural tissue properties from these more granular and higher quality *q*-space data [13] [14] [15] [16]. Neurite orientation dispersion and density imaging (NODDI) is a recently proposed multi-compartment biophysical model of brain microstructure [17] that can compute the non-collinear properties of neurite orientation dispersion index (ODI) and neurite density index (NDI), corresponding to the degree of incoherence in fiber orientations and to the intracellular volume fraction, respectively, within each imaging voxel. A special advantage of NODDI over previously proposed biophysical diffusion models is that the multi-shell HARDI imaging data required is within the current MR scanner hardware, pulse sequence and acquisition time constraints for clinical research studies [17]. An additional benefit of NODDI over DTI is that free water diffusion is isolated into a separate biophysical compartment; therefore, partial volume averaging with cerebrospinal fluid does not contaminate estimates of tissue microstructure as it often does with DTI [18] [19].

In this study, we apply NODDI to investigate how ODI and NDI change with brain maturation, with comparison to the standard DTI metrics of FA, MD, AD, and RD. We hypothesize that, given the more direct relationship between NODDI metrics and white matter microstructure, ODI and NDI will show features of human brain development not apparent from DTI. We also postulate that NODDI measures will be better correlated with chronological age for individual white matter tracts than are DTI metrics and will be a better predictor of age when used in a partial least squared regression model.

## Methods

### 2.1 Study Subjects

This study is based on diffusion imaging data from 66 healthy human subjects ages 7–63 (30 female, 36 male). A total of 70 subjects were tested and imaged, 21 at the Children’s Hospital of Philadelphia (CHOP) and the remainder within the University of California (UC) system. One subject was excluded due to excessive motion and 3 subjects were excluded as outliers based on criteria given below. Six subjects were each scanned 2–4 times for purposes of test-retest reproducibility analysis; however, data from only 5 of these subjects were used due to excessive motion in one subject.

### 2.2 Image Acquisition

MR imaging at both UC and CHOP was performed on a 3T TIM Trio MR scanner (Siemens, Erlangen, Germany) using a 32-channel phased-array radio-frequency head coil. High-resolution structural MR images of the brain were collected using an axial 3D magnetization prepared rapid acquisition gradient-echo (MPRAGE) T1-weighted sequence (TE = 1.64 ms, TR = 2530 ms, TI = 1200 ms, flip angle of 7°) with 160 1.0 mm contiguous partitions of 1x1 mm resolution on a 256x256 matrix. Whole-brain diffusion-weighted images were collected at two different diffusion-weighted sensitivities: *b* = 1000 s/mm^2^ with 30 directions, and *b* = 3000 s/mm^2^ with 64 directions. The *b* = 1000 s/mm^2^ data were used for DTI analysis, while both the *b* = 1000 s/mm^2^ and *b* = 3000 s/mm^2^ data were used for the NODDI analysis. Diffusion images at both weightings were acquired using multislice 2D single-shot spin-echo echo-planar imaging with monopolar gradients. Parallel imaging with the iPAT technique was used with a reduction factor of 2, one excitation, and 2mm interleaved axial slices with no gap at an isotropic resolution of 2x2 mm on a 128x128 matrix. The echo time (TE) and repetition time (TR) were 80ms and 10000ms for the images acquired at *b* = 1000 s/mm^2^, and 119ms and 13900ms for images acquired at *b* = 3000 s/mm^2^. Additional brain volumes were acquired with no diffusion weighting (*b* = 0 s/mm^2^), one with the TE/TR values used for the *b* = 1000 s/mm^2^ data, and two with the TE/TR values used for the *b* = 3000 s/mm^2^ data. The total acquisition time for diffusion imaging was approximately 20 minutes.

### 2.3 Structural MR Imaging Analysis

The 3D T1-weighted MPRAGE images of all subjects were examined for structural abnormalities by a board-certified pediatric neuroradiologist. Intracranial volumes (ICV), total grey matter volume, and cortical white matter volume were obtained from each subject's 3D T1 images using FreeSurfer 4.5.0 [20].

### 2.4 Diffusion Image Processing

#### 2.4.1 DTI pre-processing

FMRIB's Linear Image Registration Tool (FLIRT; www.fmrib.ox.ac.uk/fsl/flirt) was used to register all diffusion-weighted volumes to their corresponding *b* = 0 s/mm^2^ volume, and to correct for motion and eddy currents [21]. Relative displacements between consecutive diffusion volumes were calculated for each subject, including both translation and rotation, and subjects with a >2mm average displacement were excluded for excessive motion. One subject was excluded due to image artifacts, and five test-retest scans were excluded due to excessive motion in the *b* = 3000 s/mm^2^ data. This led to analysis of 70 subjects, including 5 subjects with 2–4 test-retest scans each, for a total of 14 test-retest scans.

Subsequently, the Brain Extraction Tool (BET; http://www.fmrib.ox.ac.uk/analysis/research/bet) was used to remove non-brain tissue, and FSL's DTIFIT was used to calculate maps of FA, MD, AD and RD.

#### 2.4.2. Multi-compartment Biophysical Modeling of Diffusion MR Imaging

The acquisition of diffusion data using two different diffusion weightings (b = 1000 s/mm^2^ and b = 3000s/mm^2^) allows for a complementary model of diffusion using NODDI. The NODDI code was modified to account for the differing TEs/TRs between scans acquired at *b* = 1000 s/mm^2^ and *b* = 3000 s/mm^2^ by fitting the NODDI model to the normalized diffusion-weighted images instead of the raw images. As per the developers' recommendation, the diffusion-weighted images at each *b* value were normalized by the *b* = 0 s/mm^2^ images acquired with the same TE/TR scan parameters, generating images with TE/TR-independent signal intensity. This modeling generated ODI and NDI maps for each subject.

#### 2.4.3 Tract-Based Spatial Statistics

FSL's Tract-Based Spatial Statistics (TBSS) tool [22] was used to align individual FA maps to FSL’s standard adult FA template. Following registration, the FA maps of all subjects were thinned to create white matter skeletons. Then, RD, AD, ODI, and NDI maps were created and registered using the TBSS registrations of FA to the adult FA template, and the skeleton mask was applied to the registered images.

### 2.5. Age trajectory analysis of diffusion metrics

#### 2.5.1 Modeling trajectories of regions of interest

Analyses of age trajectory over regions of interest (ROIs) were performed hierarchically, following the white matter divisions described in Simmonds et al. [23]. First, global trajectories were obtained by averaging each diffusion metric along the white matter skeleton of each subject. Three subjects had global ODI or NDI values with Z-scores above 4.5, and were excluded as outliers from all analyses, leaving a total of 66 subjects.

Next, three groups of white matter were compared; core tracts based on the JHU-DTI81 atlas [24], white matter regions adjacent to cortical grey matter regions derived from the Harvard-Oxford (HO) atlas in FSL, and white matter regions adjacent to subcortical grey matter regions also derived from the HO atlas. These regions were extracted by taking the intersection of the white matter skeleton mask from TBSS with the cortical and subcortical regions of the probabilistic HO atlas thresholded at a probability of 50%. Following Simmonds et al.'s [23] terminology, we call these three groups: core tracts, cortical regional termination zones (RTZs), and subcortical RTZs. The core tracts were then further subdivided into six groups: callosal tracts, cerebellar tracts, brainstem projection tracts, non-brainstem projection tracts, association tracts, and limbic tracts. The cortical RTZs were also further divided into five groups; prefrontal, sensorimotor, parietal, occipital, and temporal.

Four different two-parameter models were fit to each white matter group trajectory: linear, logarithmic, exponential growth, and exponential decay. Correlation coefficient (R) values for each fit were calculated to determine the best-fitting model for each parameter and for each white matter group. Three different three-parameter models were also fit to each white matter group trajectory: Poisson, quadratic, and logistic. The goodness of fit of each of these models was compared to each best-fitting two-parameter model using an F-test, which takes into account the additional degrees of freedom of the three-parameter models [25] [26].

In order to compare regional differences in trajectories, it was first recognized that ODI was generally best fit by an exponential growth model: ODI = b1 * exp(b2*age) [eq. *1]*. Meanwhile, NDI was best fit by a logarithmic growth model: NDI = b1 + b2 * ln(age) [eq. *2]*.

Equation 1 can be linearized to the form: ln(ODI) = ln(b1) + b2 * age [eq. 3]. Analysis of covariance was then performed using the linearized exponential growth model for ODI (natural log of ODI versus age), and linearized logarithmic model for NDI (NDI versus natural log of age), separately for: 1) core tracts (JHU), cortical RTZs, and subcortical RTZs; 2) the six core tract groups; and 3) the five cortical RTZ groups.

#### 2.5.2. Whole brain ODI and NDI correlations with age

To further investigate regional variation in age-related changes of ODI and NDI, the *randomise* function from FSL was used to assess for significant fits on a voxel-wise basis along the white matter skeleton. To assess for significant exponential growth fits of ODI, linear correlations of ln(ODI) versus age were used. To assess for significant logarithmic growth fits of NDI, linear correlations of NDI versus ln(age) were used. *Randomise* uses nonparametric permutation testing, and allows for cluster-level inference when the threshold-free cluster enhancement (TFCE) approach is used [27]. *Randomise* was used with two different contrasts, 5000 permutations each, to test for correlations and anti-correlations of ln(ODI) with age, and NDI with ln(age). The statistical maps for each contrast were corrected for multiple voxel-wise comparisons with TFCE using a significance threshold of p<0.05.

To create white matter skeleton maps of voxel-wise b2 values (from equations 1 and 2) for NDI and ODI, respectively representing the rate of logarithmic and rate of exponential growth, each subject's skeleton map was first smoothed using a Gaussian kernel with full-width-half-maximum (FWHM) of 9mm. The smoothing kernel was limited to the white matter skeleton in order to avoid partial volume effects. This methodology was adapted from Li et al. [28], in which FA skeleton maps were smoothed to improve signal to noise ratio for independent component analysis. ODI and NDI values from the smoothed white matter skeletons of each subject were then used for voxel-wise model fitting, and b2 maps from the exponential growth equation (eq. 1) and logarithmic growth equation (eq. 2) were calculated respectively for ODI and NDI. These b2 maps were then masked by the TFCE maps derived from *randomise*.

#### 2.5.3. Prediction of brain maturity using NODDI metrics versus DTI metrics

In order to further compare the correlation of age with NODDI metrics versus DTI metrics, partial least squares regression (PLSR) was employed separately with NODDI and DTI to create and compare models for the prediction of age. PLSR is a technique that combines features of principal component analysis (PCA) and multiple linear regression to predict a set of response variables from a set of predictor variables; this technique is particularly suited to variables with high collinearity [29]. Two different PLSR models were computed. One model used predictive variables of ODI and NDI in each of the six core tract (JHU) groups, in all JHU tracts combined, and in the global white matter skeleton, resulting in 8 regions for each ODI and NDI. The second model used predictive variables of RD and AD in all of the same white matter regions. The two PLSR models each had 16 predictive variables (2*(6+1+1)) and one response variable (age). Each variable was normalized by standard deviation, and PLSR was performed varying the number of specified PLS components from 1–10, each using 10-fold cross validation with 20 Monte-Carlo repetitions. This was considered an acceptable number of iterations since the values of the predicted root mean square error stabilized after 10 Monte Carlo runs. The predicted root mean square error for each model was plotted as a function of number of PLSR components for 1–10 components.

As a supplementary analysis, PLSR was performed in the same manner described above, but with the addition of three predictor variables from FreeSurfer segmentation to each model: intracranial volume, total grey matter volume, and cortical white matter volume.

### 2.6 Test-retest reproducibility analysis

Coefficients of variation of the diffusion metrics within each white matter group examined in section *2*.*5*.*1* were calculated using the scans of the five adult subjects with two to four repeated scans each.

## Results

### 3.1. Subject demographics

**Fig 1:**
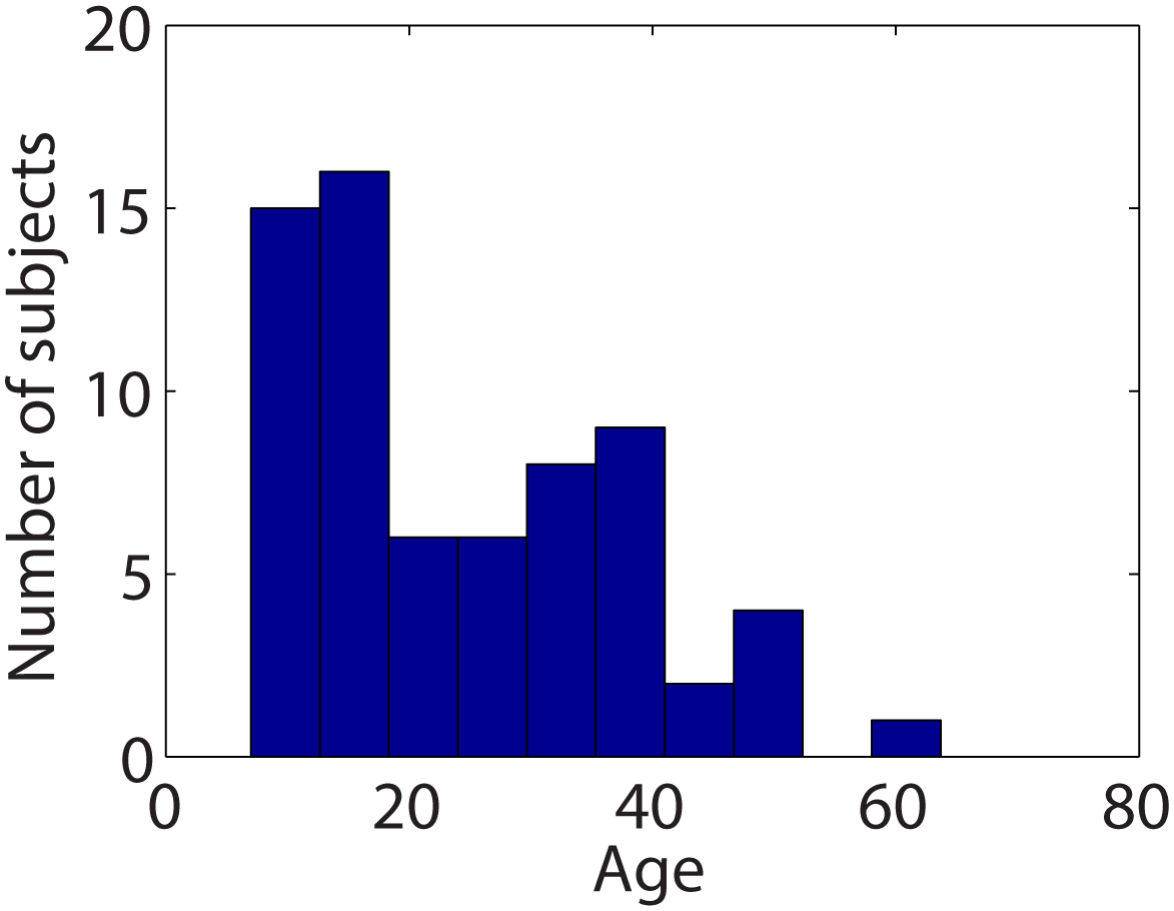
Subject age distribution.

Fig 1 displays the distribution of subject ages. The ages range from 7–63 years, and the mean subject age is 25 years, with a standard deviation of 14 years. Since this is a study of brain development, children and adolescents less than 21 years of age are overrepresented relative to adults. Table 1 displays demographic breakdown by site. The 3D structural MR scans of all subjects were noted to be free of anatomic abnormalities by a board-certified pediatric neuroradiologist.

**Table 1:** Subject demographics by site.

	# of subjects	# females	age(mean±sd)	age(median, range)
UC	47	21 (45%)	26±15	22, 7–64
CHOP	19	9 (47%)	22±10	19, 7–41
Total	66	30 (45%)	25±14	21.5,7–64

### 3.2. Region of interest trajectory modeling

**Fig 2:**
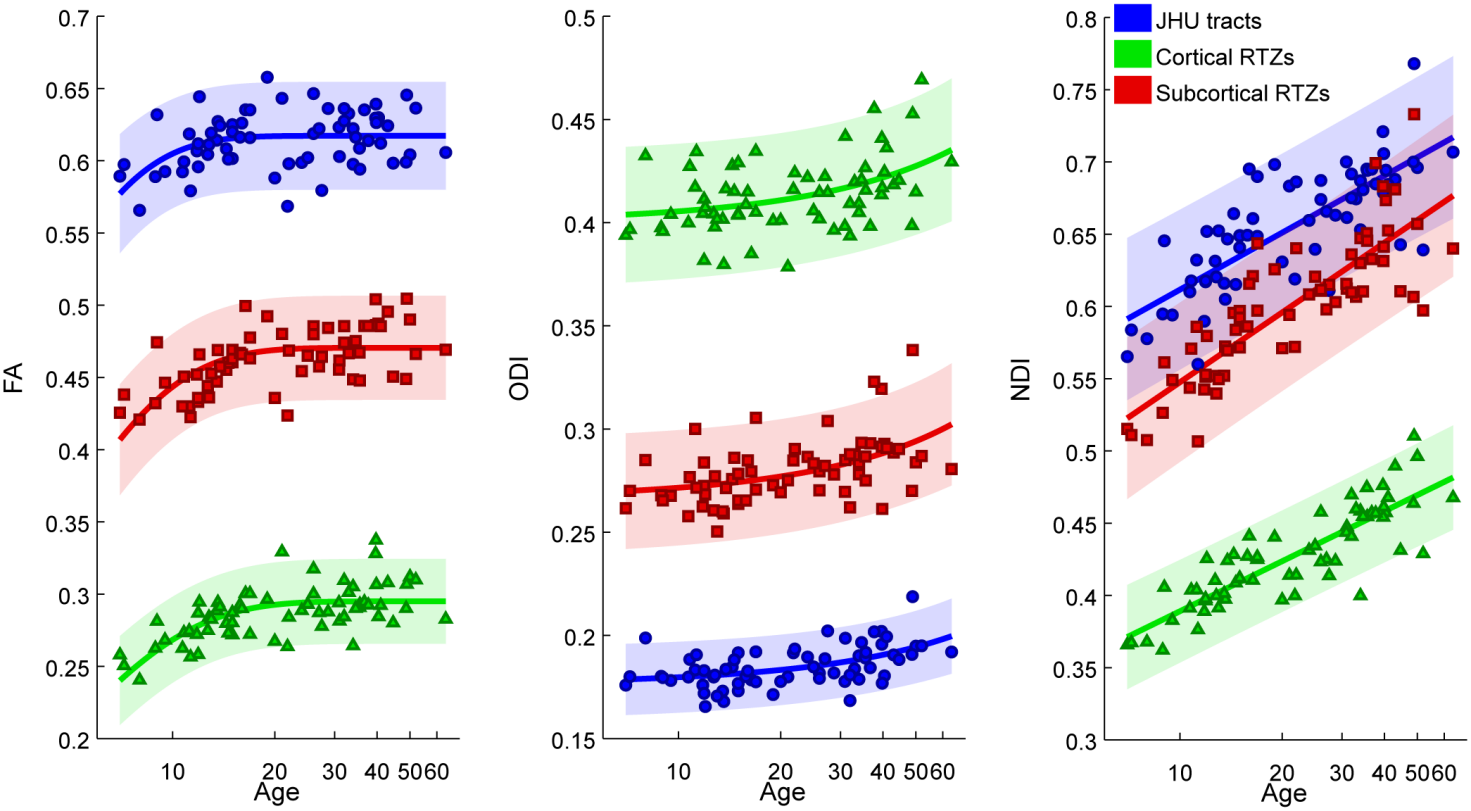
Subject FA, ODI, and NDI age trajectories in all core tracts (JHU) averaged (blue), all cortical RTZs averaged (green), and all subcortical RTZs (red). Shaded regions represent 95% confidence intervals.

**Fig 3:**
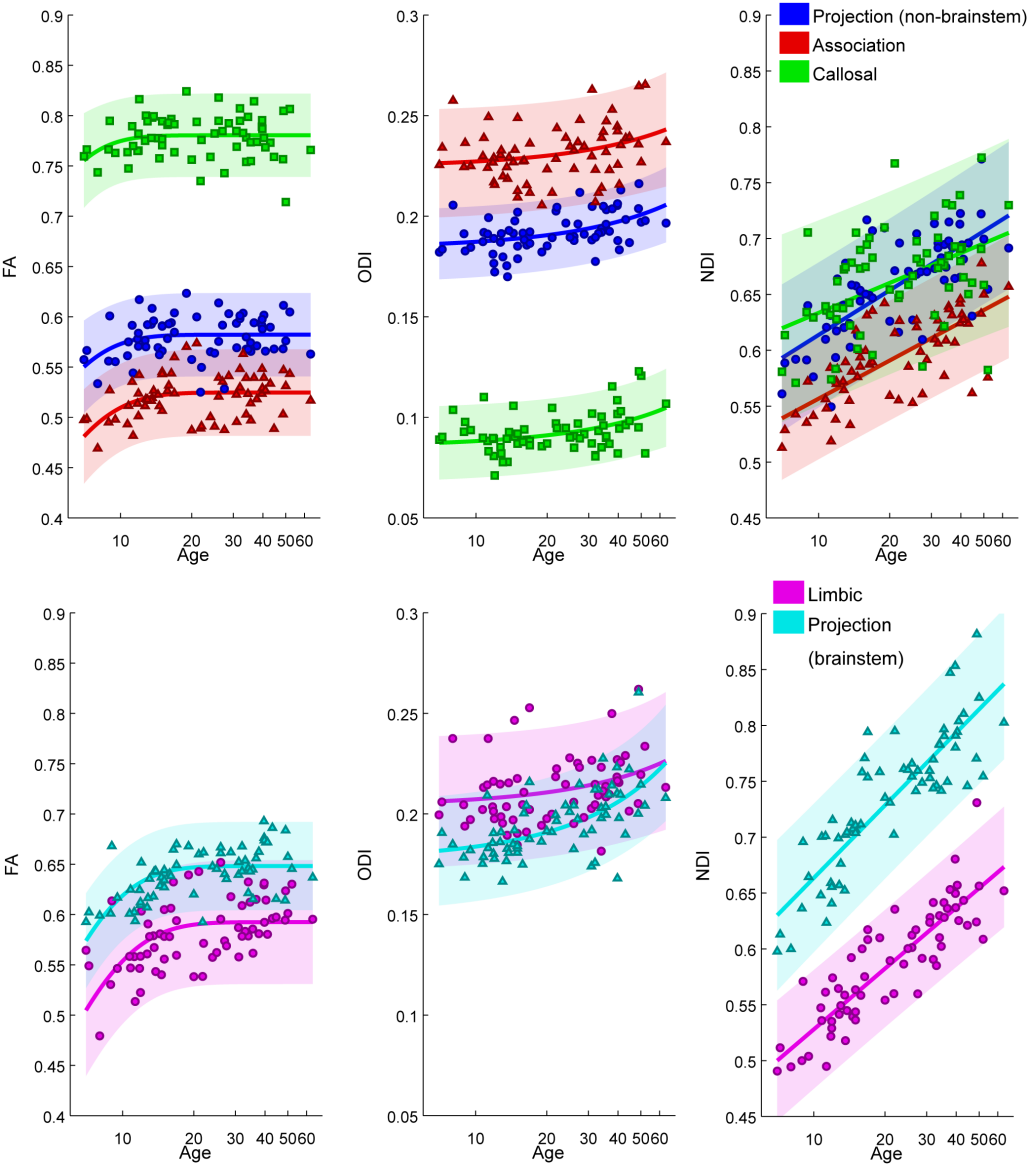
Subject FA, ODI, and NDI age trajectories in each group of JHU tracts (excepting cerebellar tracts): projection (non-brainstem, blue), association (red), callosal (green), limbic (magenta), projection (brainstem, cyan). Shaded regions represent 95% confidence intervals.

**Fig 4:**
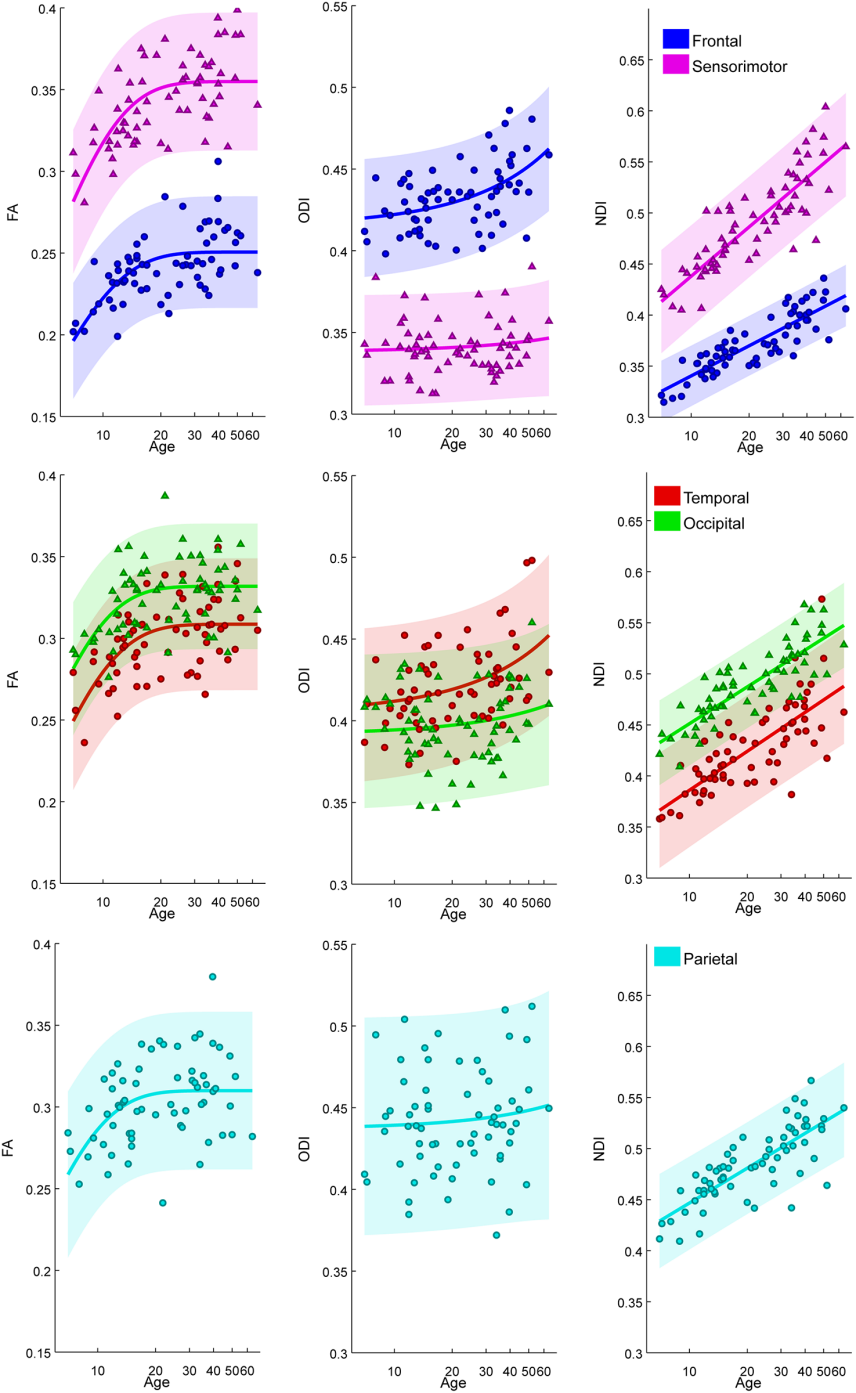
Subject FA, ODI, and NDI age trajectories in each group of cortical RTZ tracts: prefrontal (blue), sensorimotor (magenta), parietal (cyan), temporal (red), occipital (green). Shaded regions represent 95% confidence intervals.

The results of the region of interest age trajectory modeling are displayed in Figs 2–4 and Tables 2–4. In general, FA trajectories are best fit by an exponential decay model (and occasionally a logarithmic model), while ODI trajectories are best fit by an exponential growth model (and occasionally by a linear model), and NDI trajectories are best fit by a logarithmic model (Tables 2–4). The higher order fits using 3 parameters did not perform better by an F-test for any of the parameters.

**Table 2:** Fitting results for FA, ODI, and NDI in global white matter (WM), core tracts averaged, cortical RTZs averaged, and subcortical RTZs averaged.

	R (lin)	p (lin)	R (log)	p (log)	R (exp decay)	p (exp decay)	R (exp grow)	p (exp grow)
FA								
Global WM	0.327	0.0073	0.397	9.8E-04	**0.456**	1.2E-04	0.326	0.0076
JHU Tracts	0.253	0.040	0.314	0.010	**0.379**	0.0017	0.252	0.041
Cortical RTZs	0.547	2.0E-06	0.618	3.1E-08	**0.638**	8.3E-09	0.542	2.7E-06
Subcortical RTZs	0.560	1.0E-06	0.620	2.8E-08	**0.571**	5.4E-07	0.556	1.3E-06
ODI								
Global WM	0.566	7.3E-07	0.525	6.2E-06	0.232	0.060	**0.567**	7.0E-07
JHU Tracts	0.505	1.5E-05	0.468	7.5E-05	0.157	0.21	**0.505**	1.5E-05
Cortical RTZs	0.418	4.7E-04	0.379	0.0017	0.193	0.12	**0.419**	4.6E-04
Subcortical RTZs	0.490	3.0E-05	**0.501**	1.8E-05	0.312	0.011	0.488	3.3E-05
NDI								
Global WM	0.782	8.7E-15	**0.837**	0.0E+00	0.785	5.8E-15	0.774	2.6E-14
JHU Tracts	0.711	2.3E-11	**0.770**	4.3E-14	0.736	1.9E-12	0.704	4.3E-11
Cortical RTZs	0.823	0.0E+00	**0.855**	0.0E+00	0.766	7.1E-14	0.815	1.1E-16
Subcortical RTZs	0.770	4.2E-14	**0.829**	0.0E+00	0.787	4.6E-15	0.759	1.5E-13

Four different two-parameter models were used: linear, logarithmic, exponential decay, exponential growth. Models with the highest significant R values are bolded.

**Table 3:** Fitting results for FA, ODI, and NDI in each set of JHU tracts using four different two-parameter models: linear, logarithmic, exponential decay, exponential growth.

	R (lin)	p (lin)	R (log)	p (log)	R (exp decay)	p (exp decay)	R (exp grow)	p (exp grow)
FA								
All JHU	0.253	0.040	0.314	0.010	**0.379**	0.0017	0.252	0.041
Projection (non-brainstem)	0.152	0.22	0.195	0.12	**0.289**	0.018	0.152	0.22
Association	0.233	0.059	0.289	0.018	**0.376**	0.0018	0.232	0.060
Limbic	0.511	1.2E-05	**0.552**	1.6E-06	0.489	3.1E-05	0.508	1.3E-05
Projection (brainstem)	0.501	1.8E-05	**0.577**	4.0E-07	0.548	1.9E-06	0.498	2.1E-05
Callosal	0.012	9.3E-01	0.053	0.67	**0.224**	0.071	0.012	0.93
Cerebellar	0.343	0.0048	**0.399**	9.1E-04	0.357	0.0033	0.341	0.0050
ODI								
All JHU	0.505	1.5E-05	0.468	7.5E-05	0.157	0.21	**0.505**	1.5E-05
Projection (non-brainstem)	**0.470**	7.0E-05	0.462	9.6E-05	0.212	0.087	0.469	7.3E-05
Association	0.286	0.020	0.223	0.073	0.000	1.0E+00	**0.287**	0.019
Limbic	0.286	0.020	0.260	0.035	0.095	0.45	**0.287**	0.020
Projection (brainstem)	**0.613**	4.5E-08	0.614	4.2E-08	0.464	8.7E-05	0.610	5.5E-08
Callosal	0.409	6.6E-04	0.345	0.0045	0.000	1.0E+00	**0.412**	5.8E-04
Cerebellar	0.370	0.0022	0.352	0.0038	0.196	0.12	**0.370**	0.0022
NDI								
All JHU	0.711	2.3E-11	**0.770**	4.3E-14	0.736	1.9E-12	0.704	4.3E-11
Projection (non-brainstem)	0.659	1.8E-09	**0.722**	8.0E-12	0.704	4.2E-11	0.652	3.1E-09
Association	0.680	3.3E-10	**0.729**	3.8E-12	0.695	9.8E-11	0.675	5.0E-10
Limbic	0.827	0.0E+00	**0.866**	0.0E+00	0.793	2.1E-15	0.817	1.1E-16
Projection (brainstem)	0.791	2.7E-15	**0.856**	0.0E+00	0.839	0.0E+00	0.780	1.2E-14
Callosal	0.428	3.3E-04	0.480	4.5E-05	**0.485**	3.7E-05	0.425	3.7E-04
Cerebellar	0.751	3.6E-13	**0.783**	8.1E-15	0.671	6.8E-10	0.746	6.5E-13

**Table 4:** Fitting results for FA, ODI, and NDI in each set of cortical RTZs using four different two-parameter models: linear, logarithmic, exponential decay, exponential growth.

	R (lin)	p (lin)	R (log)	p (log)	R (exp decay)	p (exp decay)	R (exp grow)	p (exp grow)
FA								
All Cortical RTZs	0.547	2.0E-06	0.618	3.1E-08	**0.638**	8.3E-09	0.542	2.7E-06
Prefrontal	0.537	3.4E-06	**0.593**	1.5E-07	0.590	1.8E-07	0.530	4.7E-06
Sensorimotor	0.582	3.0E-07	**0.634**	1.1E-08	0.608	6.3E-08	0.576	4.3E-07
Temporal	0.471	6.5E-05	0.528	5.1E-06	**0.550**	1.7E-06	0.467	7.8E-05
Occipital	0.315	0.010	0.399	9.2E-04	**0.502**	1.8E-05	0.311	0.011
Parietal	0.308	0.012	0.379	0.0017	**0.415**	5.3E-04	0.304	0.013
ODI								
All Cortical RTZs	0.418	4.7E-04	0.379	0.0017	0.193	0.12	**0.419**	4.6E-04
Prefrontal	**0.493**	2.6E-05	0.476	5.3E-05	0.294	0.017	**0.493**	2.6E-05
Sensorimotor	0.105	0.40	0.040	0.75	0.000	1.0E+00	0.106	0.40
Temporal	**0.402**	8.3E-04	0.391	0.0012	0.270	0.028	**0.402**	8.3E-04
Occipital	0.164	0.19	0.061	0.63	0.000	1.0E+00	0.165	0.18
Parietal	0.093	0.46	0.064	0.61	0.075	0.55	0.094	0.45
NDI								
All Cortical RTZs	0.823	0.0E+00	**0.855**	0.0E+00	0.766	7.1E-14	0.815	1.1E-16
Prefrontal	0.827	0.0E+00	**0.859**	0.0E+00	0.766	6.5E-14	0.820	0.0E+00
Sensorimotor	0.832	0.0E+00	**0.853**	0.0E+00	0.766	6.9E-14	0.825	0.0E+00
Temporal	0.732	2.9E-12	**0.758**	1.8E-13	0.680	3.5E-10	0.724	6.2E-12
Occipital	0.793	2.1E-15	**0.831**	0.0E+00	0.749	4.6E-13	0.785	5.9E-15
Parietal	0.738	1.5E-12	**0.786**	5.3E-15	0.732	3.0E-12	0.731	3.3E-12

Results from this analysis for the subjects scanned at UC alone are also presented in S1 Fig and S1 Table to demonstrate that the results do not differ when considering only one site.

#### 3.2.1 Comparison of JHU tracts, cortical RTZs, and subcortical RTZs

Fig 2 illustrates that the core JHU tracts exhibit the highest values of FA, followed by subcortical RTZs, while cortical RTZs have the lowest values. Reciprocally, the cortical RTZs exhibit the highest values of ODI, followed by subcortical RTZs, while the JHU tracts have the lowest values of ODI. Finally, similar to FA, the JHU tracts demonstrate the highest values of NDI, followed by subcortical RTZs, while the cortical RTZs have the lowest values of NDI (Fig 2). Cortical RTZs have a slower rate of logarithmic growth in NDI (higher b2 in eq. 2) relative to subcortical RTZs and JHU tracts, while subcortical RTZs show a faster rate of logarithmic growth in NDI relative to cortical RTZs and JHU tracts (Table 5).

**Table 5:** Fitting results and regional difference analysis of JHU tracts, cortical RTZs, and subcortical RTZs using exponential growth fit for ODI, and logarithmic growth fit for NDI.

	R	p (reg)	b2	p (slope diff)
ODI (exp growth fit)				
Global WM	0.57	**6.2E-07**	0.00178	-
JHU Tracts	0.50	**1.4E-05**	0.00196	0.55
Cortical RTZs	0.42	**4.7E-04**	0.00132	0.18
Subcortical RTZs	0.49	**2.2E-05**	0.00200	0.45
NDI (log fit)				
Global WM	0.84	**1.2E-18**	0.0607	-
JHU Tracts	0.77	**2.7E-14**	0.0569	0.65
Cortical RTZs	0.85	**3.4E-20**	0.0500	**0.039**
Subcortical RTZs	0.83	**4.7E-18**	0.0697	**0.012**

#### 3.2.2 Comparison of JHU tracts by tract type

Fig 3 reveals that, of the JHU tracts, the callosal tracts demonstrate the highest values of FA and the lowest values of ODI. The brainstem projection tracts have the highest values of NDI, with callosal and non-brainstem projection tracts also showing high values of NDI. The association and limbic tracts have the lowest values of FA, highest values of ODI, and lowest values of NDI (Fig 3). Table 6 shows that the brainstem projection tracts demonstrate a higher rate of exponential growth in ODI (higher b2 in eq. 1) relative to the other JHU tract groups: non-brainstem projection, association, limbic, callosal, and cerebellar. The brainstem projection tracts and limbic tracts show a higher rate of logarithmic growth in NDI relative to the other JHU tract groups, while the callosal and association tracts show a lower rate of logarithmic growth in NDI relative to the other JHU tract groups.

**Table 6:** Fitting results and regional difference analysis of JHU tract groups using exponential growth fit for ODI, and logarithmic growth fit for NDI.

	R	p (reg)	b2	p (slope diff)
ODI (exp growth fit)				
All JHU	0.50	**1.4E-05**	0.00196	-
Projection (non-brainstem)	0.47	**6.4E-05**	0.00174	0.42
Association	0.28	**0.021**	0.00126	0.082
Limbic	0.29	**0.017**	0.00164	0.31
Projection (brainstem)	0.61	**3.5E-08**	0.00380	**0.0056**
Callosal	0.40	**8.5E-04**	0.00321	0.16
Cerebellar	0.37	**0.0021**	0.00188	0.51
NDI (log fit)				
All JHU	0.77	**2.7E-14**	0.0569	-
Projection (non-brainstem)	0.72	**5.5E-12**	0.0577	0.37
Association	0.73	**2.6E-12**	0.0493	**0.024**
Limbic	0.87	**3.0E-21**	0.0785	**0.014**
Projection (brainstem)	0.86	**2.8E-20**	0.0937	**1.2E-06**
Callosal	0.48	**3.9E-05**	0.0386	**8.1E-05**
Cerebellar	0.78	**5.0E-15**	0.0616	0.79

#### 3.2.3 Comparison of cortical RTZs by region

Fig 4 shows that, of the cortical RTZ regions, the sensorimotor white matter exhibits the highest values of FA, the lowest values of ODI, and the highest values of NDI. The prefrontal white matter demonstrates the lowest values of FA and NDI. The prefrontal and parietal white matter have the highest values of ODI. ODI in the sensorimotor, occipital, and parietal white matter are not fit significantly by an exponential growth model (or any other two-parameter model), as ODI in these areas remains relatively constant over the age range examined. The sensorimotor white matter shows a higher rate of logarithmic growth in NDI relative to the other RTZ regions, while the prefrontal tracts demonstrate a lower rate of logarithmic growth in NDI (Table 7).

**Table 7:** Fitting results and regional difference analysis of cortical RTZs using exponential growth fit for ODI, and logarithmic growth fit for NDI.

	R	p (reg)	b2	p (slope diff)
ODI (exp growth fit)				
All Cortical RTZs	0.42	**4.7E-04**	0.00132	-
Prefrontal	0.49	**2.8E-05**	0.00169	0.14
Sensorimotor	0.11	0.39	3.83E-04	0.18
Temporal	0.40	**8.0E-04**	0.00174	0.11
Occipital	0.16	0.19	7.18E-04	0.52
Parietal	0.09	0.46	5.14E-04	0.28
NDI (log fit)				
All Cortical RTZs	0.85	**3.4E-20**	0.0500	-
Prefrontal	0.86	**1.3E-20**	0.0425	**0.0091**
Sensorimotor	0.85	**4.6E-20**	0.0695	**2.4E-04**
Temporal	0.76	**1.2E-13**	0.0550	0.75
Occipital	0.83	**3.3E-18**	0.0520	0.70
Parietal	0.79	**3.3E-15**	0.0493	0.31

### 3.3. Voxel-wise rate-of-change of NODDI metrics

**Fig 5:**
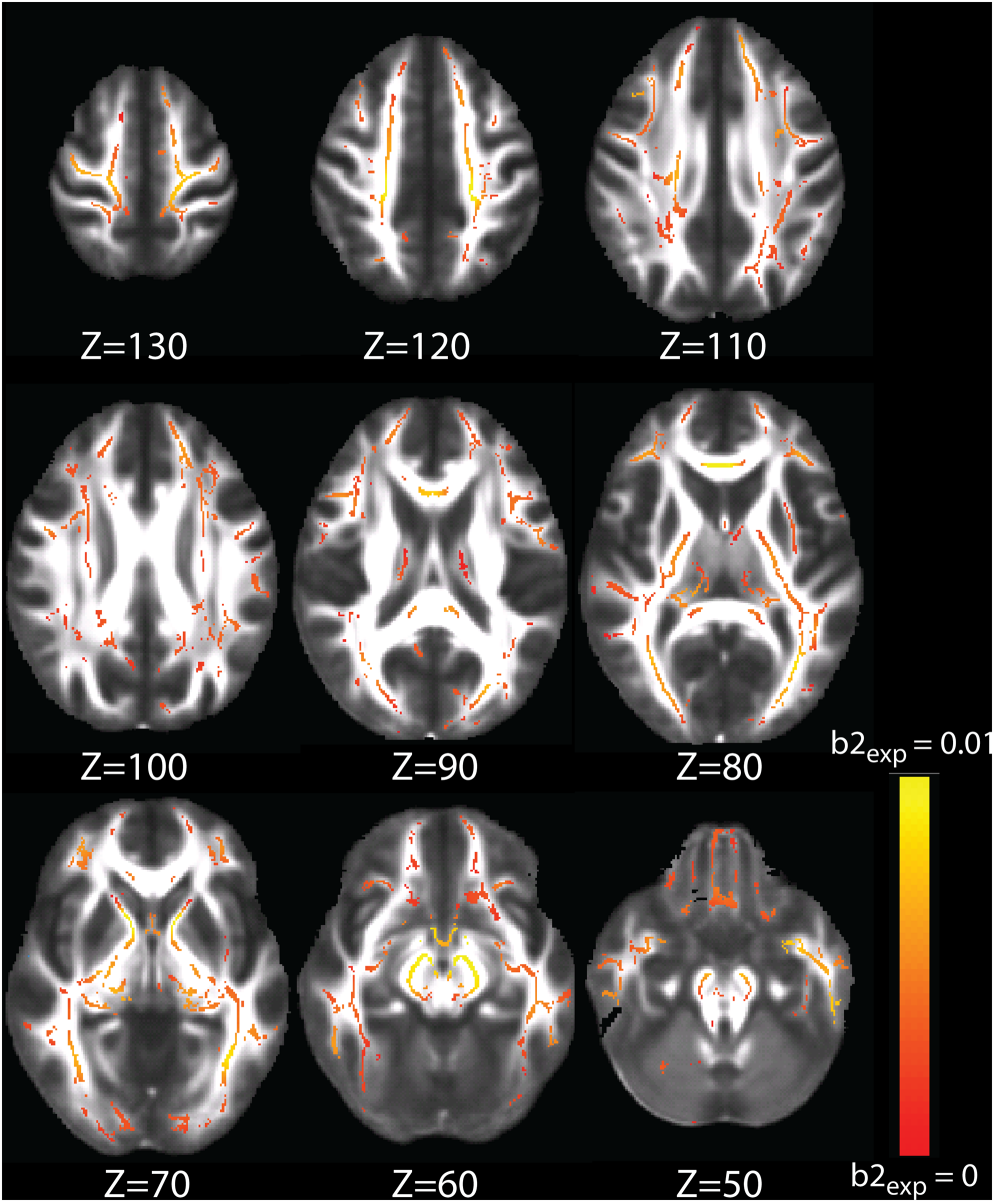
ODI exponential growth b2 map (eq. 1) plotted on a red-yellow color scale, where colored voxels are significantly fit by an exponential model (with TFCE-correction) and yellow represents higher b2.

**Fig 6:**
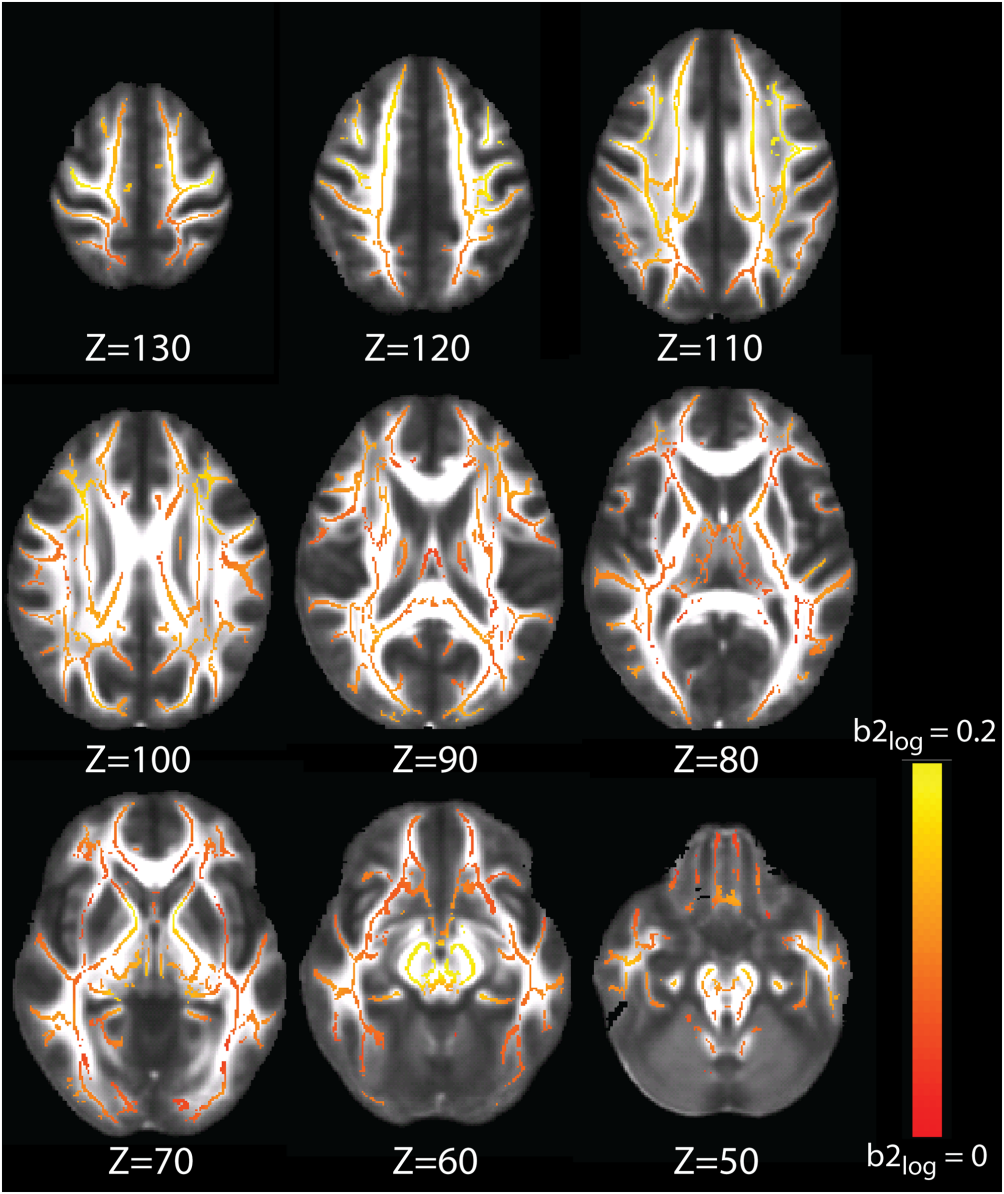
NDI logarithmic growth b2 map (eq. 2) plotted on a red-yellow color scale, where colored voxels are significantly fit by a logarithmic model (with TFCE-correction) and yellow represents higher b2.

The ODI and NDI b2 maps (from eq.1 and eq.2, respectively) are displayed in Figs 5 and 6, respectively. The ODI map confirms some of the results seen from the region of interest ODI exponential growth trajectory results: the brainstem and genu of the corpus callosum (GCC) demonstrate higher rates of exponential ODI growth, reflecting the results from the ROI analyses of heightened rates of exponential ODI growth in the brainstem projection tracts and the trend towards heightened rates of exponential ODI growth in the callosal tracts. The ODI b2 maps additionally exhibit more extensive exponential growth in the posterior limbs of the internal capsules as compared with the anterior limbs.

As expected from the goodness of fit results of the logarithmic model for NDI in the regions of interest, the NDI b2 map exhibits a much higher number of voxels with TFCE-corrected significant fits as compared with the ODI b2 map. The NDI map confirms results from the region of interest NDI logarithmic growth trajectory results: the brainstem and sensorimotor regions show higher rates of logarithmic growth of NDI, while the GCC and prefrontal regions show lower rates of growth.

### 3.4. Prediction of brain maturity using NODDI metrics versus DTI metrics

**Fig 7:**
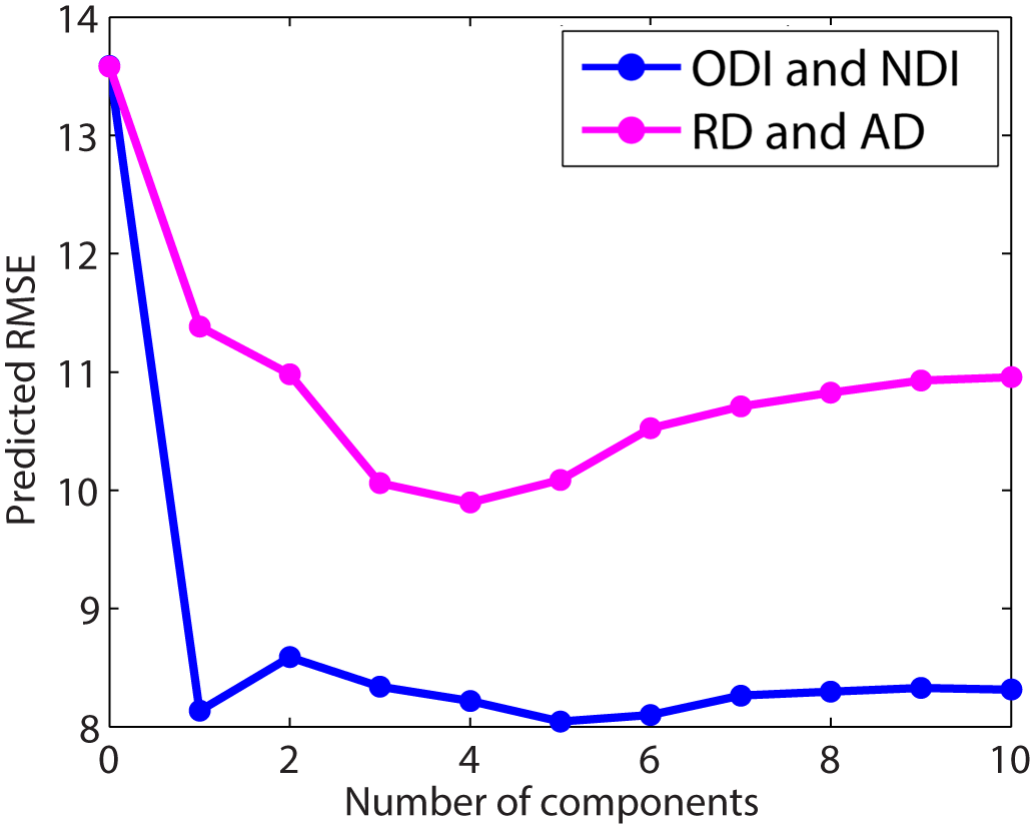
Predicted root mean square error of the estimation of age using a partial least squares (PLS) model constructed using ODI and NDI compared to the PLS model constructed using RD and AD.

The predicted RMSE (PRMSE) as a function of number of PLS components is displayed in Fig 7 for both the ODI and NDI model as well as the RD and AD model. For the ODI and NDI model, PRMSE is effectively minimized at a value of 8.1 years using the first PLS component. For the RD and AD model, PRMSE is minimized at a value of 9.9 years using the first four PLS components.

The PRMSE results from the supplementary analysis, which included additional predictor variables of intracranial volume, total grey matter volume, and white matter volume, are displayed in S2 Fig. At one PLS component, the PRMSE of the ODI and NDI model decreases from 8.1 years to 8.0 years, indicating that these additional variables contribute little predictive value to the NODDI model. At four PLS components, the PRMSE of the RD and AD model decreases from 9.9 to 9.1, indicative of a stronger contribution of the additional variables to the predictive value of the DTI model. However, the error in the DTI model remains larger than in the NODDI model.

### 3.5 Test-retest reproducibility

**Fig 8:**
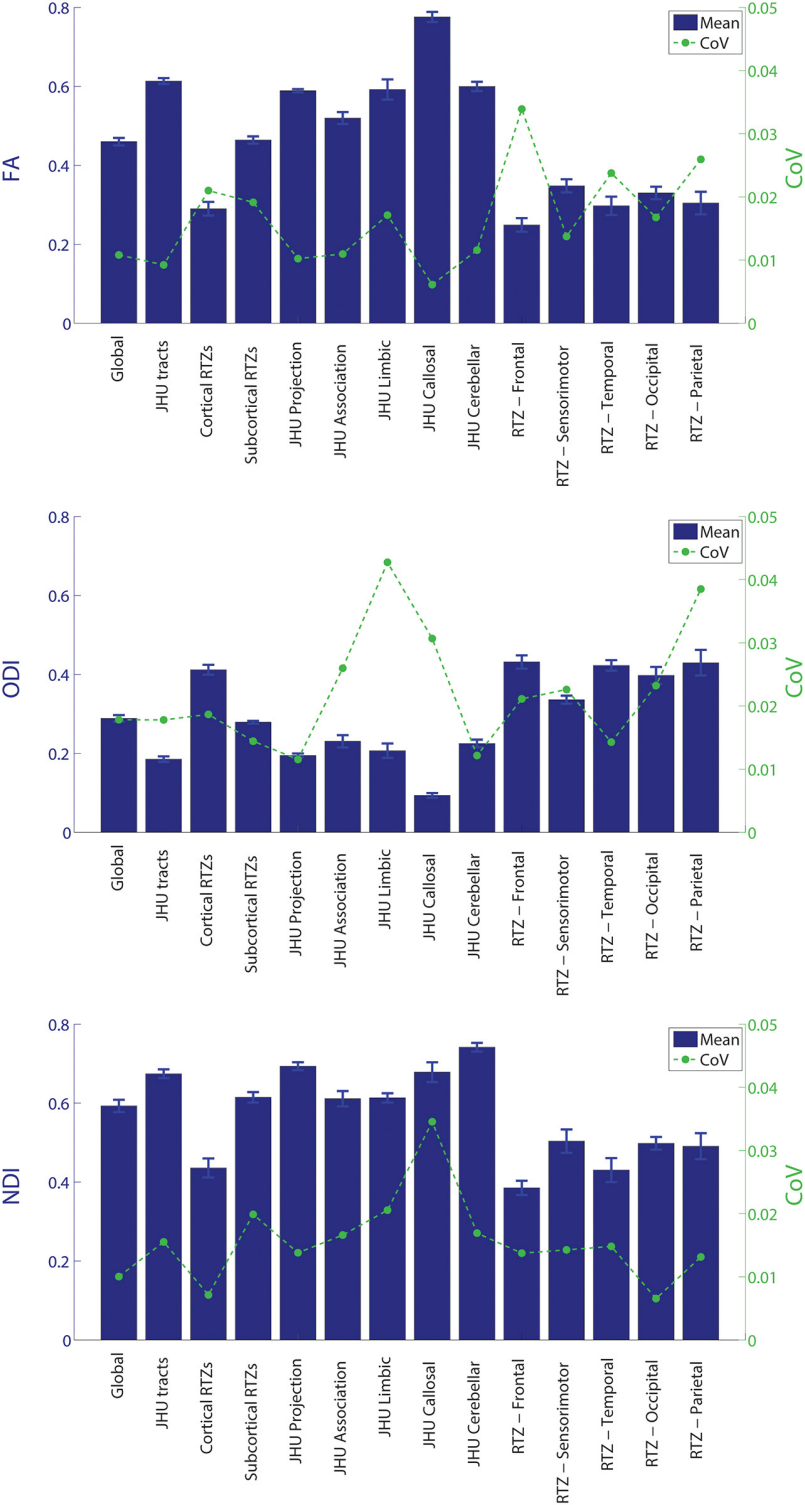
Test-retest reliability results for FA, ODI, and NDI.

The means and coefficients of variation of ODI and NDI within each examined white matter group are displayed in Fig 8. The coefficients of variation (CoVs) of the ODI and NDI values within white matter groups are comparable to those of the FA values, with all CoVs below 5%, indicating little variation in any of the three metrics.

## Discussion

### 4.1. Developmental trajectories of Neurite Density Index and Fiber Orientation Dispersion Index

This is the first study to characterize maturational changes of neurite density (NDI) and fiber orientation dispersion (ODI) in human white matter from childhood to middle age. We find that NDI exhibits striking increases over this age range following a logarithmic growth pattern, while ODI also rises but follows an exponential growth pattern. These results reveal that, while neurite density index increases rapidly in childhood and more slowly in adulthood, fiber orientation dispersion index increases more slowly in childhood, and accelerates in adulthood. This is a novel finding that suggests a more biologically specific interpretation of the well-established age-related changes of FA over the lifespan that show decelerating growth during childhood and adolescence, a plateau during early adulthood, followed by accelerating decay after the fourth decade of life [30]. Our data demonstrate that the rise of FA during the first two decades of life is dominated by increasing NDI, with little change in ODI to counteract it. Although the oldest ages of the human lifespan are not represented in our sample, extrapolation of our results suggests that the fall in FA during later adulthood is due to an exponential rise of ODI that overcomes the slowing increase of NDI. This is supported by the fact that ODI is a stronger determinant of FA than is NDI [17], as also shown in S3 Fig.

To our knowledge, the only other study to investigate NODDI metrics in the typically developing human brain explores these metrics in newborns [31]. Kunz et al. [31] compared NODDI values in white matter regions of interest in 13 infants at term. As expected, the general relationships between FA, ODI, and NDI demonstrated in our results match those in Kunz et al., showing that white matter regions with relatively higher FA demonstrate relatively higher NDI and relatively lower ODI (Section 3.2). Kunz et al. [31] also show clear differentiation between the anterior and posterior limbs of the internal capsules, which they attribute to the respectively absent myelin and partial myelination in these regions (at term); our results suggest that these regions continue to exhibit different developmental trajectories into adulthood (Figs 5 and 6).

The trend of increasing FA during development has previously been attributed to axonal density, myelination, and/or increases of fiber diameter [9] [32] [33] [34]. Meanwhile, histological studies have suggested a diversity of processes in aging that could cause decreasing FA; these include accumulation of water-containing balloons in myelin sheaths [35] [36], accumulation of redundant myelin, thickening and/or splitting of myelin lamella, loss of small nerve fibers [37] [38], shortening of internodes, and/or alterations of fiber diameter [39]. In fact, there has been evidence of continued remodeling of myelin into the sixth decade of life [40] [41]. Our results could explain the white matter changes over age as observed with DTI metrics, where increases of FA (and decreases of MD and RD) during development are dominated by increases of NDI, and decreases of FA with senescence are driven by the accelerating increases of ODI. Thus, our results point to increases in fiber diameter and myelination in development, two processes which are difficult to distinguish using diffusion MR imaging, but both of which would lead to increased NDI, which represents the intracellular volume fraction, as well as the physiological effect of increased axonal conduction speed [42]. Due to the underrepresentation of subjects over the age of 40 in our study cohort, we do not aim to characterize the effects of aging. However, the previously proposed factors of redundant myelin, ballooning of myelin sheaths, and splitting/thickening of the myelin lamella are consistent with our observation of exponentially rising ODI with age during adulthood, as well as continuing increases in NDI. Furthermore, Westlye et al. [43] found peak global FA at approximately 29 years of age, while total white matter volume did not peak until around 50 years. This and the evidence of myelin remodeling in late adulthood is concordant with our result of prolonged increases of NDI. It is alternatively possible that the observed increases of NDI with age are caused not by changes of axonal density, but by glial proliferation in aging brain [44]. Still, we cannot exclude the possibility of an eventual fall of NDI with senescence.

In addition to characterizing ODI and NDI developmental trajectories, our results demonstrate the superiority of NODDI over DTI in explaining variance of chronological age. The R values for models of NDI over age are consistently significantly higher for NDI than for models of FA over age (Tables 2–4). Additionally, our PLSR results show higher predictive power of the NODDI metrics compared to DTI metrics for age estimation (Fig 7), indicating better quantification of the effect of age on white matter. The supplementary PLSR analysis (S2 Fig) further demonstrates that NODDI explains variance of chronological age independently of the covariates, and potential confounds, of intracranial volume, total grey matter volume, and white matter volume. In contrast, the DTI metrics, for which predictive power improves significantly in conjunction with these covariates, may be confounded in part by partial volume effects from differences in head size.

### 4.2. Regional variations

The regional variations that we observe, both in the region-of-interest and voxel-wise analyses, reflect anatomic differences in NDI and ODI trajectories over age. Using our present best-fit models—logarithmic growth for NDI and exponential growth for ODI—it is complicated to exactly specify the timing of development or aging. While a larger dataset with an extended age range of subjects might provide the power necessary to implicate higher-order models, the models in use are highly compelling, especially for NDI, for which fits give rise to correlation coefficient values consistently in the range of 0.72–0.87 (Tables 2–4). Furthermore, while it is difficult to describe timing using these models, we nonetheless find results that are concordant with studies investigating region-specific changes to white matter over the lifespan using DTI.

In our b2 maps (Figs 5 and 6), we see high rates of exponential growth of ODI in the genu of the corpus callosum (GCC) compared to sparse and small growth in the splenium of the corpus callosum (SCC); in contrast, NDI shows sparse logarithmic growth in the GCC, compared to more extensive and larger logarithmic growth in the SCC. These observations are consistent with findings in literature that show larger increases of FA in the SCC than the GCC during development, and earlier and more significant decline of FA in the GCC than in the SCC [33] [45] [46]. In the SCC, the significant growth of NDI coupled with limited increase of ODI would result in more prolonged and significant increases of FA, compared to the GCC, whereas limited growth of NDI coupled with significant growth of ODI would lead to smaller and less prolonged increases of FA.

Our b2 maps reveal extensive exponential growth of ODI in the posterior limbs of the internal capsules, but not the anterior limbs, while NDI exhibits comparable logarithmic growth in both the anterior and posterior limbs. Imperati et al. [45] used k-means clustering of DTI to separate white matter voxels based upon age trajectories, and found that the anterior and posterior limbs differentiated from one another across all cluster solutions, i.e., solutions with different numbers of specified clusters. They further found that the anterior limbs of the internal capsules and the cerebral peduncles exhibited the greatest increases of FA during development, and the highest preservation of FA in adulthood. This finding is consistent with our observations in the anterior limbs of internal capsules, where the insignificant exponential growth of ODI coupled with prolonged growth of NDI would result in the preservation of FA in adulthood. While the cerebral peduncles in our ODI b2 maps demonstrate high rates of exponential growth, they also show the highest rates of logarithmic growth in NDI, which is consistent with the significant increases of FA that Imperati et al. [45] report during development. Using group difference analysis of different age groups, Qiu et al. [46] found decreases of MD in both the anterior and posterior limbs of the internal capsules for young adults compared to adolescents, but found increases of FA of young adults compared to adolescents only in the anterior portions of the internal capsule. These results lend further credence to our observations in the internal capsules, where the similar growth of NDI in the anterior and posterior limbs would cause similar decreases of MD in these two regions, but accelerating growth of ODI in the posterior limbs would have a primary effect of slowing, stopping, and then reversing increases of FA.

In our regional difference analyses of the JHU core tracts, we find higher rates of logarithmic growth of NDI in the limbic tracts and the brainstem projection tracts (Fig 3, Table 3). While the brainstem projection tracts also demonstrate higher rates of growth of ODI, the limbic tracts do not. Our findings in the limbic tracts are well-corroborated by other studies that find prolonged development in the hippocampus and temporal regions [30]. Westlye et al. [43] find the steepest developmental curves in the dorsal cingulum bundle, and protracted development in the parahippocampal cingulum bundles. In fact, with 430 subjects aged 8–85 years of age, they do not find age-related decreases of FA at all in the parahippocampal cingulum bundles. While we do not find that the temporal RTZs exhibit significantly different NDI or ODI growth trajectories from other cortical RTZs, our RTZ results should be interpreted with more caution, as these regions comprise very peripheral white matter and are more sensitive to errors in the registration required to perform TBSS. Finally, prior studies find that projection fibers tend to mature earlier than association fibers [47]. Lebel et al. [30] find that commissural and projection fibers mature earliest, while association fibers continue to mature at later ages, and frontal-temporal connections demonstrate the most prolonged development. While our finding of higher rates of logarithmic growth of NDI in the brainstem projection tracts may initially seem to run counter to existing literature, these results could be reconciled by our observation that ODI also exhibits higher rates of growth than in other tracts. It is therefore feasible that the behavior of FA in the brainstem projection tracts becomes driven more strongly by the increases of ODI than those of NDI earlier in life due to the high acceleration of ODI growth in these tracts relative to other tracts.

### 4.3. Test-retest reproducibility

The ODI and NDI values within different white matter groups show excellent test-retest reproducibility, with all tracts and parameters having coefficients of variation (CoVs) below 5%, and the majority with CoVs below 3%. This reproducibility is consistent with a previous report which shows comparable CoVs between DTI and NODDI in global white matter [48]. A caveat to our reproducibility results is that all of our repeat scans are in adults in their 20s; these results are therefore not representative of the full age range of our subjects.

### 4.4 Study limitations

A main limitation of this study is the smaller number of subjects in the older range, resulting in a bias of age-related trajectories towards development. The effect of this bias is evident in our modeled trajectories for FA, where higher order quadratic and Poisson fits did not perform better by an F-test than the exponential decay fit. The trajectories of NDI and particularly ODI in aging therefore require replication as well as better characterization over the entire lifespan.

Another limitation is the differing acquisition TEs/TRs of the diffusion images acquired at the two different b values. Caution might therefore be needed in comparing the absolute magnitudes of our NODDI parameters to values obtained using different acquisition parameters. Nonetheless, our acquisition parameters are consistent within our study cohort, and deviations from ideal acquisition parameters would be expected to blur age-related relationships, as opposed to producing false ones.

### 4.5. Future directions

There are numerous directions for future research of white matter development and aging using NODDI, with starting points provided by the wealth of DTI studies of white matter changes over the human lifespan. For example, Mishra et al. [49] have used the DTI microstructural correlation method [50] to examine the inter-tract dependencies of FA during childhood brain maturation. Furthermore, multivariate data-driven approaches such as independent component analysis [28] or k-means clustering [45] can be employed to identify developmentally correlated white matter at the voxel level. Used in conjunction with NODDI, these methods could potentially yield even more valuable information than with DTI, since the NODDI metrics are more directly related to the underlying white matter microstructure. Additionally, tractographic approaches to studying white matter maturation can be applied in each subject’s native space, as atlas-based voxel-wise techniques suffer from errors in registration that may be exacerbated when studying subjects over broad age ranges. Alternatively, registration in TBSS can be improved using techniques such as groupwise atlas with tensor-based registration [51]; this would be particularly important in a study including an older age range of subjects in which atrophy would be a much greater effect.

It could be of further use to relate structural brain changes using NODDI to functional changes during development. There are concordant patterns of brain development elucidated using structural and functional MRI; as local functional connectivity weakens within the cortex, cortical grey matter thins, and as long-range white matter tracts increase in FA, long-range functional connectivity increases [1]. Nonetheless, a study which investigates both structural and functional connectivity in childhood brain development found that only some changes of functional connectivity had structural correlates [52]. It is possible that NODDI could provide greater insight into the structural-functional relationship, both within or outside the context of development.

It would also be valuable to investigate grey matter development using the NODDI metrics of isotropic (CSF) volume fraction, NDI, and ODI, which translate naturally to grey matter, unlike DTI metrics. In grey matter, ODI provides a marker of grey matter complexity, quantifying the pattern of sprawling dendritic processes [17].

Also of great value would be a longitudinal study to identify more individually specific maturational trajectories of NDI and ODI, with correlation to neuropsychological assessments to link these age-related changes to the development of higher cognitive functions. Ultimately, these efforts would contribute to the goals of characterizing how changes in brain networks during development give rise to high-level human cognition, as a prerequisite to understanding the neural underpinnings of atypical cognitive development in neuropsychiatric disorders.

## Supporting Information

S1 Fig UC-only analysis: Subject FA, ODI, and NDI age trajectories in all core tracts (JHU) averaged (blue), all cortical RTZs averaged (green), and all subcortical RTZs (red). Shaded regions represent 95% confidence intervals.
(TIF)

S2 Fig Predicted root mean square error of the estimation of age using a partial least squares (PLS) model constructed using ODI and NDI compared to the PLS model constructed using RD and AD, with the additional variables (to each model) of intracranial volume, total grey matter volume, and cortical white matter volume. (TIF)

S3 Fig Scatter plot of NDI versus ODI, colored by FA, for every voxel along the mean white matter skeleton across all study subjects. (TIF)

S1 Table UC-only fitting results for FA, ODI, and NDI in global white matter (WM), core tracts averaged, cortical RTZs averaged, and subcortical RTZs averaged. Four different two-parameter models were used: linear, logarithmic, exponential decay, exponential growth.
(DOCX)
