# Supplementary figures and images for: White Matter Changes of Neurite Density and Fiber Orientation Dispersion during Human Brain Maturation
Source: PLoS One. 2015 Jun 26;10(6):e0123656. doi: 10.1371/journal.pone.0123656 (PMC4482659; doi:10.1371/journal.pone.0123656)

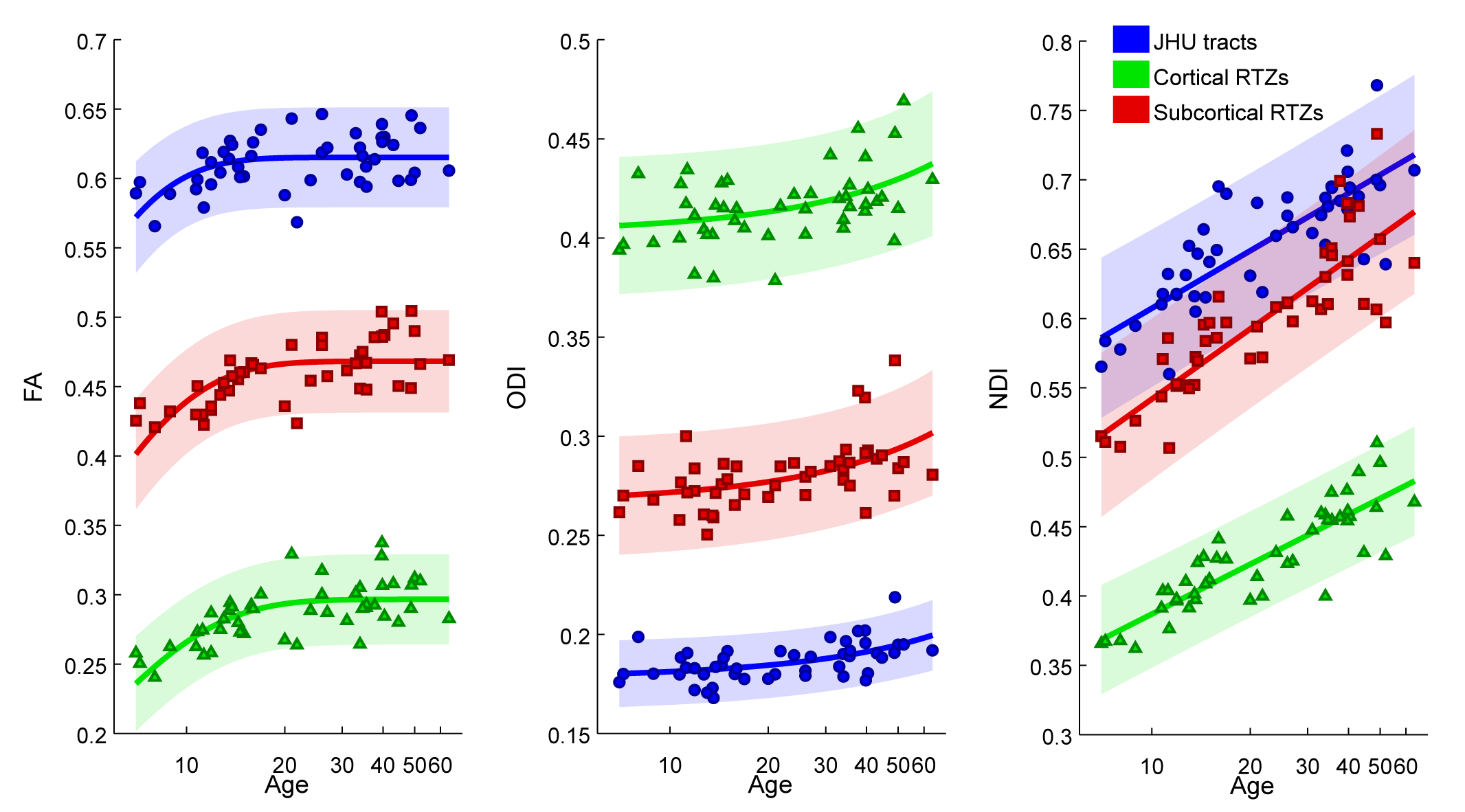

Supplement: S1 Fig — Shaded regions represent 95% confidence intervals. (TIF) [file pone.0123656.s001.tif]

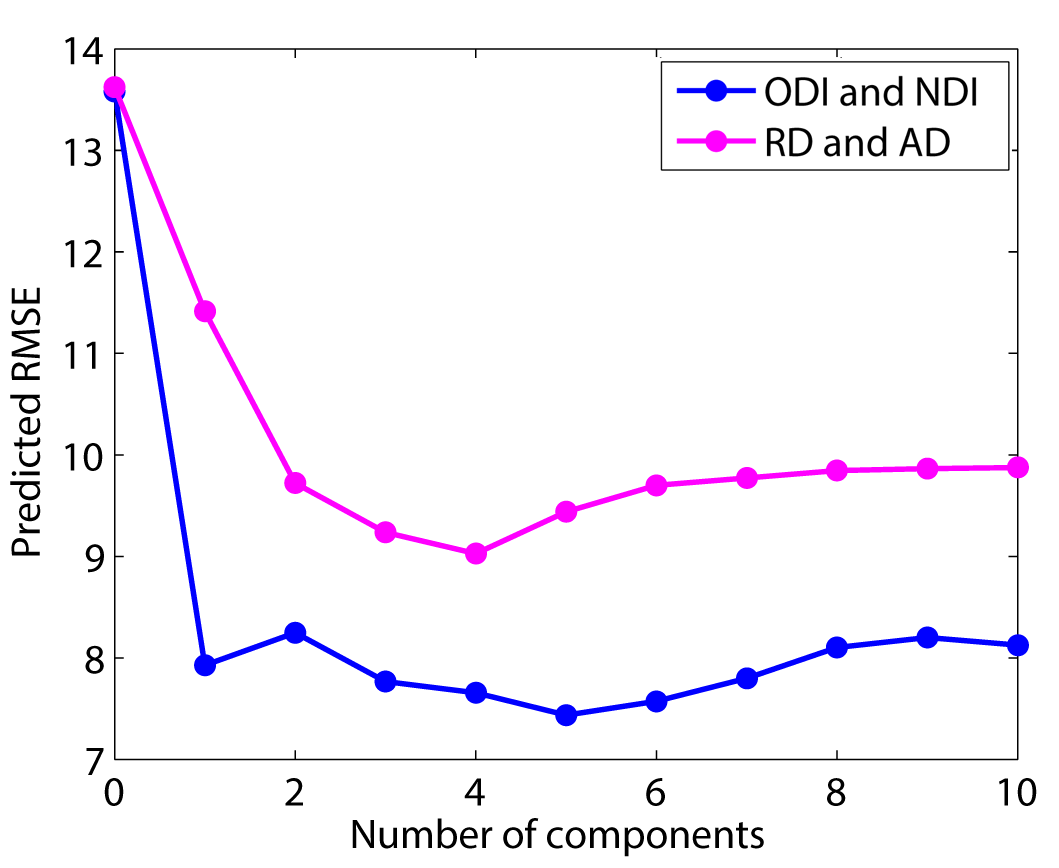

Supplement: S2 Fig — (TIF) [file pone.0123656.s002.tif]

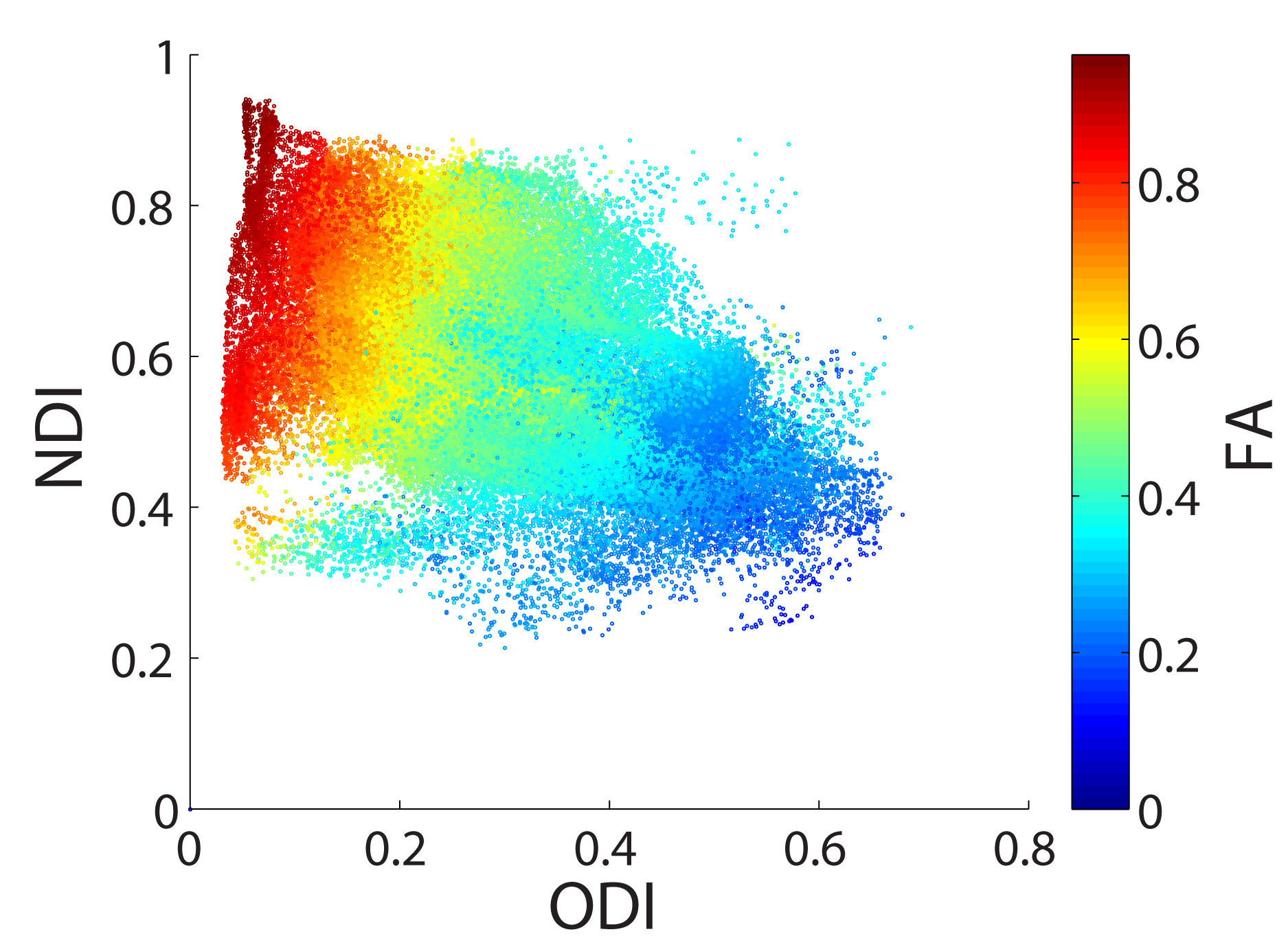

Supplement: S3 Fig — (TIF) [file pone.0123656.s003.tif]
