# Supplementary material for: White Matter Changes of Neurite Density and Fiber Orientation Dispersion during Human Brain Maturation
Source: PLoS One. 2015 Jun 26;10(6):e0123656. doi: 10.1371/journal.pone.0123656 (PMC4482659; doi:10.1371/journal.pone.0123656)
Supplement: S1 Table — Four different two-parameter models were used: linear, logarithmic, exponential decay, exponential growth. (DOCX) [file pone.0123656.s004.docx]

Table S1. UC-only fitting results for FA, ODI, and NDI in global white matter (WM), core tracts averaged, cortical RTZs averaged, and subcortical RTZs averaged. Four different two-parameter models were used: linear, logarithmic, exponential decay, exponential growth.

|  | R (lin) | p (lin) | R (log) | p (log) | R (exp decay) | p (exp decay) | R (exp grow) | p (exp grow) |
| --- | --- | --- | --- | --- | --- | --- | --- | --- |
| FA |  |  |  |  |  |  |  |  |
| Global WM | 0.411 | 0.0041 | 0.486 | 5.3E-04 | **0.513** | 2.3E-04 | 0.409 | 0.0036 |
| JHU Tracts | 0.361 | 0.013 | 0.429 | 0.003 | **0.447** | 0.0016 | 0.360 | 0.011 |
| Cortical RTZs | 0.567 | 3.2E-05 | 0.647 | 8.8E-07 | **0.672** | 2.4E-07 | 0.560 | 2.1E-05 |
| Subcortical RTZs | **0.653** | 6.6E-07 | 0.707 | 2.7E-08 | 0.612 | 4.9E-06 | 0.648 | 5.1E-07 |
| ODI |  |  |  |  |  |  |  |  |
| Global WM | 0.593 | 1.1E-05 | 0.554 | 5.4E-05 | 0.258 | 0.080 | **0.593** | 9.1E-06 |
| JHU Tracts | 0.524 | 1.6E-04 | 0.489 | 4.9E-04 | 0.176 | 0.24 | **0.524** | 1.3E-04 |
| Cortical RTZs | 0.435 | 2.2E-03 | 0.409 | 0.0043 | 0.229 | 0.12 | **0.436** | 2.1E-03 |
| Subcortical RTZs | 0.497 | 3.8E-04 | **0.502** | 3.2E-04 | 0.279 | 0.058 | 0.495 | 2.8E-04 |
| NDI |  |  |  |  |  |  |  |  |
| Global WM | 0.798 | 1.8E-11 | **0.856** | 1.8E-14 | 0.807 | 7.2E-12 | 0.789 | 1.8E-11 |
| JHU Tracts | 0.739 | 3.1E-09 | **0.801** | 1.3E-11 | 0.768 | 3.1E-10 | 0.732 | 2.8E-09 |
| Cortical RTZs | 0.824 | 1.1E-12 | **0.862** | 6.8E-15 | 0.786 | 6.0E-11 | 0.816 | 9.0E-13 |
| Subcortical RTZs | 0.793 | 3.1E-11 | **0.848** | 5.7E-14 | 0.789 | 4.3E-11 | 0.782 | 1.8E-11 |
